# Effects of Unilateral Muscle Fatigue on Thermographic Skin Surface Temperature of Back and Abdominal Muscles—A Pilot Study

**DOI:** 10.3390/sports10030041

**Published:** 2022-03-08

**Authors:** Carlo Dindorf, Eva Bartaguiz, Elena Janowicz, Michael Fröhlich, Oliver Ludwig

**Affiliations:** Department of Sports Science, Technische Universität Kaiserslautern, 67663 Kaiserslautern, Germany; carlo.dindorf@sowi.uni-kl.de (C.D.); eva.bartaguiz@sowi.uni-kl.de (E.B.); ele-j@web.de (E.J.); michael.froehlich@sowi.uni-kl.de (M.F.)

**Keywords:** infrared thermography, trunk muscles, back muscles, muscle soreness, muscle fatigue

## Abstract

The present study aimed to assess the effects of asymmetric muscle fatigue on the skin surface temperature of abdominal and back muscles. The study was based on a pre-post/follow-up design with one group and included a total of 41 subjects (22 male, 19 female; age, 22.63 ± 3.91; weight, 71.89 ± 12.97 kg; height, 173.36 ± 9.95). All the participants were asked to perform side bends in sets of 20 repetitions on a Roman chair until complete exhaustion. The pre-, post- and follow-up test (24 h after) skin surface temperatures were recorded with infrared thermography. Subjective muscle soreness and muscle fatigue were analyzed using two questionnaires. The results of the post hoc tests showed that skin temperature was statistically significantly lower in the post-tests than in the pre- and follow-up tests, but no meaningful differences existed between the pre- and follow-up tests. Asymmetric side differences were found in the post-test for the upper and lower areas of the back. Differences were also noted for the front in both the upper and lower areas. No thermographic side asymmetries were found at the pre- or follow-up measurement for either the back or the front. Our results support the potential of using thermographic skin surface temperature to monitor exercise and recovery in athletes, as well as its use in rehabilitational exercise selection.

## 1. Introduction

Near-infrared thermography has recently found its way into sports science issues. Numerous studies have confirmed that changes in skin temperature occur after muscular load [1,2,3,4]. This effect is of great interest in training science as well as presumably of medical importance to prevent injuries due to muscular overload [3,5,6,7].

After muscle loading, the first effects can be observed after a few minutes. However, over the course of 24 to 48 h, the thermoregulatory effects seem to balance out again [8,9,10]. Nevertheless, the study situation in this context is heterogeneous. While no clear correlations between muscle fatigue and thermoregulatory effects have been found [10,11], there certainly seem to be correlations in physiologically measurable parameters, such as electromyography and muscle strength [2,12,13,14]. Likewise, infrared measurement of skin temperature does not appear to be able to predict delayed onset muscle soreness (DOMS) 24 h after exercise [15].

It is of particular interest whether muscular imbalances are reflected in different temperature distributions. In this case, near-infrared thermography would be a great diagnostic tool. However, the study situation is not clear. Some research groups found correlations between skin temperature and muscular strength balance [14,16], but others could not confirm this [17,18]. Most studies regarding sports science issues deal with the thermographic effects of arm and leg muscles. To the best of the authors’ knowledge, studies on the effect of unilateral fatigue of trunk muscles on surface temperature are not available to date. Therefore, the research objective of the present study was to examine the effects on the lower abdominal and back muscles. The aim of the study was to determine (1) whether asymmetric load of the lateral trunk muscles leads to thermographically visible unilateral changes in skin temperature of the lower back and trunk muscles and (2) whether possible changes are detectable to the same extent immediately after exercise and 24 h later.

## 2. Materials and Methods

For answering our research questions, the sample size was determined according to G*Power (version 3.1.9.2, Heinrich Heine University Dusseldorf, Germany) [19] with a power (1 − β) set to 0.85 (α = 0.05; f = 0.25) resulting in a minimum of 31 subjects. To take into account possible dropouts, data were collected from a total of 41 subjects (22 male, 19 female); anthropometric data of the sample are presented in Table 1. The study was approved by the ethical committee of the Technische Universität Kaiserslautern (ethics protocol number: 43; date of approval: 25 November 2021) and met the criteria of the Declaration of Helsinki [20]. All participants signed an informed consent form for the study, including permission to publish the results of the study.

The study was conducted according to the Delphi checklist of thermographic imaging in sports and exercise medicine [21]. Subjects were asked to come in a rested state without performing intense activities 48 h prior to the study. In addition, subjects were advised not to take any drugs (e.g., caffeine, alcohol) 48 h prior and not to consume large amounts of foods or water 2 h prior to the study. After the removal of the upper body clothing (females were allowed to wear a sports bra), the subjects underwent an acclimatization period of 10 min in a sitting position. The room temperature was set to 20 °C. Further environmental factors were controlled (relative humidity = 40%; wind speed = 0 ms^−1^; solar radiation = 0 Wm^−2^). Body fat and body composition were measured prior to the pre-test measurements during acclimatization period using an InBody 770 measuring device (InBody Europe, Eschborn, Germany).

**Figure 1:**
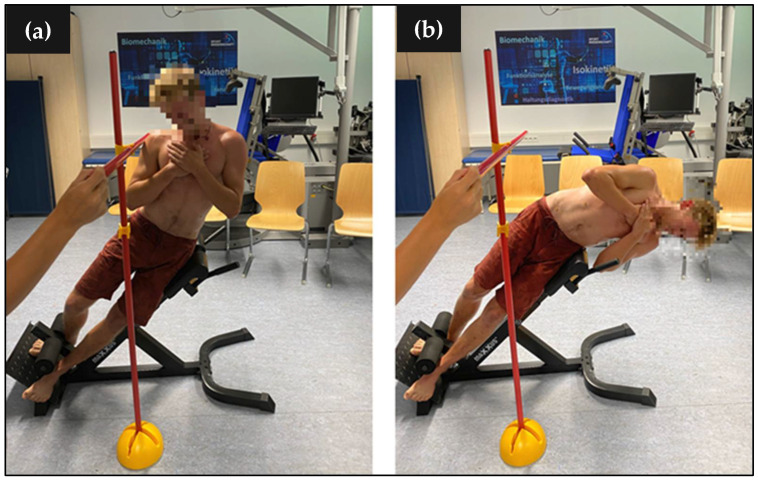
Treatment for introducing unilateral muscle fatigue through performing side bends on a Roman chair. (**a**) time point of maximal lateral flexion; (**b**) time point of maximal lateral extension.

For introducing unilateral muscle fatigue, side bends on a Roman chair were performed in sets of 20 repetitions. The side contralateral to the handedness side of each subject was targeted by the treatment. Range of motion was controlled by tactile feedback using contact points to a bar in the point of maximal lateral trunk flexion (Figure 1). The time for an upward and downward movement of one second each was given by a metronome. The treatment was performed until exhaustion where the termination criteria were defined as:

1. Across three sets, the range of motion could not fully be maintained until the last repetition (tactile feedback);
2. Execution was inaccurate across at least the last five repetitions of a set;
3. The specified number of 20 repetitions could not be achieved over 3 sets;
4. Exertion score of at least 8 on the OMNI scale [22].

Thermographic data were recorded on the back and the abdomen three times on two different days. Thermographic data were recorded and analyzed using the NEC TVS-200 ISS camera (NEC Avionics Infrared Technologies Co., Ltd., Yokohama, Japan) and the software InfReC Analyzer NS9500 Lite. It was ensured that possible sweat was removed from the subjects as soon as it occurred. Therefore, if necessary, after each set and after termination of the treatment as well as approximately every two minutes before the thermographic measurements, a dry paper towel was used to remove the sweat. For better detection of anatomical landmarks and the definition of areas of interest, cork markers (platelets with diameter 10 mm) were placed on Th12, both SIPS (spina iliaca posterior superior (SIPS), the right and left arcus costalis, and both cristae iliacae on each subject. Cork platelet locations were marked on the skin with a waterproof pen to ensure the same position for the measurement the next day. The first measurement was performed before the treatment (pre); the second, similar to [23], 10 min after the treatment (post). The third measurement was performed approximately 24 h afterwards (follow-up).

**Figure 2:**
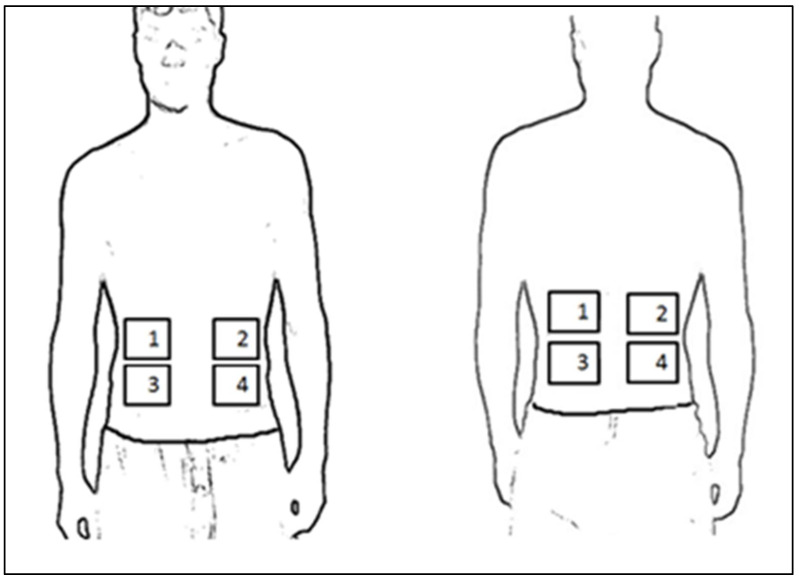
Schematic representation of the defined areas of a right-handed person. Left representation: area 1 = upper treatment side abdomen; area 2 = upper non-treatment side abdomen; area 3 = lower treatment side abdomen; area 4 = lower non-treatment side abdomen. Right representation: area 1 = upper treatment side back; area 2 = upper non-treatment side back; area 3 = lower treatment side back; area 4 = lower non-treatment side back.

For analyzing the thermographic image data, four rectangular areas were defined in the frontal and dorsal view with the help of the previously attached cork markers (see Figure 2). The mean skin temperature was calculated for each area per image and used for the further calculations. To allow a comparison of the left-handed subjects’ thermographic surface data, the data of the left-handed subjects were inverted to enable a comparison with the right-handed subjects. Therefore, area 1 and area 3 map the characteristics of the body side that the treatment was targeting (area 1 = upper part, area 3 = lower part).

Of the initial 41 subjects, the data of 33 subjects were used for the final analysis. Subjects were excluded from the study if any of the following criteria were detected: (a) the subject was not able to perform the treatment according to the instructions; (b) the subject had to terminate the treatment prematurely; (c) the sports bra was covering up a relevant area of the thermographic image; (d) subjects were not able to be present at the three measuring times.

For consideration of subjective components, the Profile of Mood States (POMS) questionnaire [24] (dimension fatigue) was additionally filled out by the participants after the treatment. Furthermore, DOMS was measured on day 2 and 3 using a seven-point Likert scale as described in [25].

To check whether there were general differences in the skin surface temperature for both abdomen and back over the three measuring time points (pre, post, follow-up), as well as the regarded areas (area 1–4), repeated-measures MANOVA was calculated. Body fat was included as covariate as it was an important influencing factor [26]. As the repeated-measures MANOVA is an omnibus test, the follow-up analysis focused on the two aspects (1) and (2) which were mentioned in the introduction. (1) For comparison of possible asymmetric differences between the body side targeted by the treatment versus the contralateral side for the three measuring times, a dependent t-test for paired samples was calculated. (2) To check changes in skin surface temperature for the measurement times, repeated-measures ANOVA was used. Mauchly tests were used to check sphericity. Greenhouse–Geisser adjustments were made to correct for violations of sphericity. No outliers had to be removed because values were all within three times the interquartile range for the variables considered. Shapiro–Wilk tests confirmed that data were normally distributed. Further necessary requirements were checked and can be assumed. To take into account the multiple comparisons problem, Bonferroni correction was performed during post hoc testing. Adjusted *p*-values were reported and computed with an alpha level of 0.05. Partial eta square (η_p_^2^) was used to report the effect size. Calculations were performed using SPSS Statistics (version 16, SPSS Inc., Chicago, IL, USA).

## 3. Results

The participants performed on average 7.27 ± 4.74 sets until exhaustion and gave a score of 8.88 ± 1.01 on the OMNI scale after the treatment. DOMS on day 2 was an average of 3.78 ± 1.33, which was lower than on day 3 which was 5.05 ± 1.10. According to the average summative score of the fatigue dimension of POMS, fatigue was rated the highest for the treatment day (19.67 ± 7.41) followed by 48 h after the treatment (16.34 ± 6.78) and 24 h after the treatment (13.20 ± 6.98).

**Figure 3:**
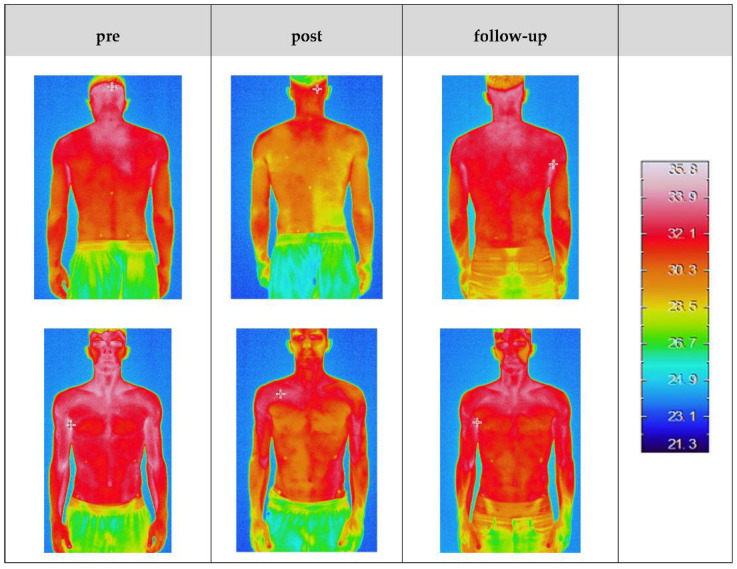
Exemplary thermographic image data of one subject for the three measurement conditions. In the subject, the muscles of the right side of the lower trunk were fatigued by side bends to the right. Significant temperature differences can be seen in the middle figures (post-test).

Table 2 shows the skin surface temperatures for the measured areas for the treatment and non-treatment sides during the three measuring times. Figure 3 shows the thermographic image data of one subject. For the image recorded directly after the treatment, a temperature difference between the left and right body side is visible.

The results of the repeated-measures MANOVA show that differences in the combined dependent variables (skin surface temperature for abdomen and back) between the measuring time points (pre, post, follow-up; F(4, 122) = 9.32, *p* < 0.001, *n* = 33, η_p_^2^ = 0.23) and regarded areas (area 1–4; F(6, 184) = 8.87, *p* < 0.001, *n* = 33, η_p_^2^ = 0.22) exist. Furthermore, there is an interaction between the measuring time points and regarded areas (F(12, 370) = 12.24, *p* < 0.001, *n* = 33, η_p_^2^ = 0.28). Therefore, the effect of the regarded areas on the combined skin surface temperature for the abdomen and the back is related to the measuring time points. Controlling the covariate body fat for the respective interaction shows that body fat influences the combined skin surface temperature for the abdomen and back (F(12, 370) = 4.85, *p* < 0.001, *n* = 33, η_p_^2^ = 0.13). In the following, the follow-up results for the aspects of interest are reported.

**Figure 4:**
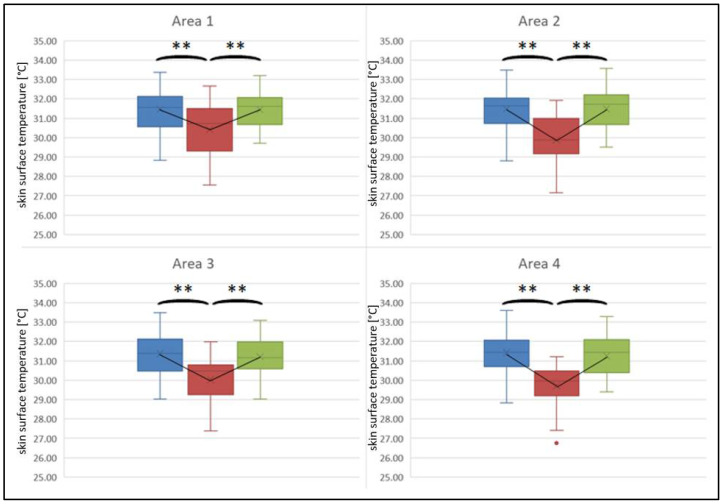
Changes in skin surface temperature of the back for both treatment side (areas 1 + 3) and contralateral side (areas 2 + 4). Blue = pre-test, red = post-test, green = follow-up test. *p*-values were adjusted according to Bonferroni; ** *p* < 0.001.

**Figure 5:**
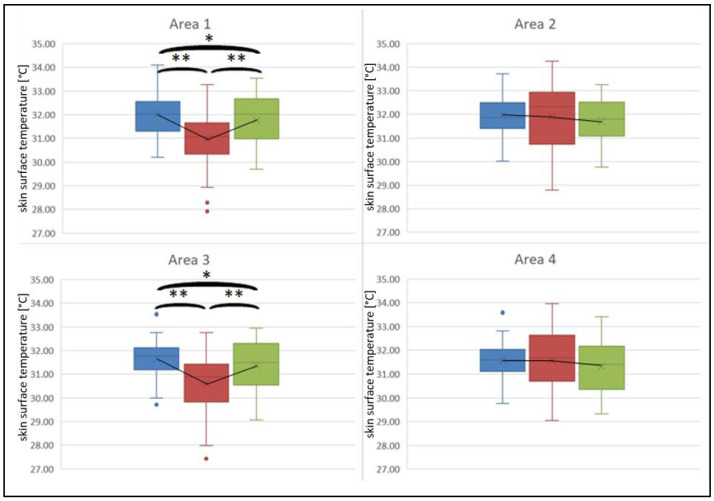
Changes in skin surface temperature of the ventral trunk for both treatment side (areas 1 + 3) and contralateral side (areas 2 + 4). Blue = pre-test; red = post-test; green = follow-up test. *p*-values were adjusted according to Bonferroni; ** *p* < 0.001, * *p* < 0.05.

Figure 4 and Figure 5 visualize the changes in skin surface temperature for the measurement areas. Comparison of the measurement areas for the three measurement times for the back shows the same characteristics for all considered areas: statistically significant differences in the three conditions could be found (area 1: F(1.71, 54.59) = 25.36, *p* < 0.001, *n* = 33, η_p_^2^ = 0.44; area 2: F(2, 64) = 65.10, *p* < 0.001, *n* = 33, η_p_^2^ = 0.67; area 3: F(2, 64) = 39.76, *p* < 0.001, *n* = 33, η_p_^2^ = 0.55; area 4: F(2, 64) = 69.49, *p* < 0.001, *n* = 33, η_p_^2^ = 0.69). The post hoc tests showed that skin temperature was statistically significantly lower in the post-tests than in the pre- and follow-up tests. However, skin temperature in the pre- and follow-up tests was not different.

Looking at the ventral side, no statistically significant differences were found for the skin temperature of the non-treatment side for the three measuring times (area 2: F(1.51, 48.16) = 1.69, *p* = 0.200, *n* = 33, η_p_^2^ = 0.05; area 4: F(1.63, 52.25) = 1.97, *p* = 0.158, *n* = 33, η_p_^2^ = 0.06). For the treatment side, statistically significant differences were found with lower temperature values in the post-tests (area 1: F(1.58, 50.53) = 27.16, *p* < 0.001, *n* = 33, η_p_^2^ = 0.46; area 3: F(1.62, 51.93) = 28.26, *p* < 0.001, *n* = 33, η_p_^2^ = 0.47). Post hoc tests showed that the thermographic values were different from each other for all of the times. Visually, however, the differences between the pre- and follow-up tests are hardly visible.

Thermographic asymmetric side differences were found for the post measurement between the upper areas (t = 5.34, *p* < 0.001, *n* = 35, r = 0.68) as well as between the lower areas (t = 2.92, *p* = 0.006, *n* = 35, r = 0.45) of the back. For the abdominal muscles, side differences were found for the upper (t = −7.29, *p* < 0.001, *n* = 34, r = 0.78) as well as the lower (t = −6.62, *p* < 0.001, *n* = 34, r = 0.75) areas. For the pre-test and follow-up test no thermographic side asymmetries were present for either back or front (*p* > 0.05). No difference was present for the comparison of the upper and lower areas of each body side for both abdominal and back muscles (*p* > 0.05).

## 4. Discussion

The results show that skin temperature was statistically significantly lower in post-tests than in pre- and follow-up tests, but no meaningful differences existed between pre- and follow-up tests. Asymmetric side differences were found in the post-test for the upper and lower areas of the back. Differences were also noted for the front in both the upper and lower areas. No thermographic side asymmetries were found at pre- and follow-up measurement for either the back or the front.

Near-infrared thermography can only measure the surface temperature of the body. While an increase in muscle blood flow and also muscle temperature is known in muscular fatigue, the measurable surface temperature largely depends on thermoregulatory effects such as skin blood flow or sweat gland activity. Our study showed significant effects in the trunk and abdominal muscles. To the best of our knowledge, there has been no study to date that investigated asymmetric thermographic effects of the lateral trunk muscles. The study results show that after unilateral muscular exercise, thermographically significant asymmetric changes in skin temperature were detectable in the lateral abdominal muscles on the loaded side. This indicates that, consistent with the muscle fatigue reported by the subjects, the treatment resulted in a change in skin blood flow in the stressed areas where a temperature decrease was observed. No changes in skin temperature could be measured on the unloaded side.

The changes on the loaded side were measurable immediately after muscular fatigue (post-test) as shown in other studies [27] and decreased after 24 h (follow-up test). This is in line with the literature, where it was reported that skin surface temperature reaches the initial state after 48 h at the latest [8]. Other research reports a much faster return to baseline skin temperatures [28]. It seems that already after 24 h, as in our case, a temperature similar to the initial state was reached. Contrarily, the delayed muscle soreness measured by means of the DOMS–Likert scale was rated the highest 48 h after the treatment, which could be related to the potential systemic inflammatory responses. Consequently, as also reported by [9], a skin temperature increase 48 h after the treatment might present. In our study, thermographic data for that time point were not measured. Therefore, there are uncertainties in the temporal development of the temperature course for the period of 48 h after the treatment, as to which further research is required.

For the back muscles, asymmetric skin surface temperatures were only present for the post-test, where the treatment side showed a slightly higher temperature compared to the non-treatment side. The post-tests showed statistically significant different temperatures compared to the pre- and follow-up tests for both the treatment and, surprisingly, the non-treatment sides. Steward et al. [9] found a similar effect and showed that after exhausting exercises of the knee flexor and extensor muscles, the anterior thigh skin temperature was statistically increased 24 h after the exercise, in both the exercised and non-exercised leg. They explained these results with a potential systemic inflammatory response 24 h after the DOMS-inducing exercises.

Another explanation could possibly be found in a cross-training effect. Escamilla-Galindo and colleagues [29] found an acute contralateral effect on skin temperature during unilateral training for the untrained limb, which also depended on the training level of the subjects. They measured the skin temperature 30 and 60 min after exercise. Low-trained participants showed greater effects than the highly trained. Based on their results, they assumed that the cross-training effect lasted at least 60 min. These differences in thermoregulatory processes could be due to physical condition and the fact that well-trained individuals delay the rise in temperature and also dissipate heat more quickly than the low-trained [30].

Lateral flexion of the trunk is performed in the abdominal region by the m. obliquus and m. quadratus lumborum and in the dorsal region by the mm. multifidii, iliocostalis lumborum, and the mm. intertransversarii, located between the vertebral transverse processes. These deep-lying muscle parts are difficult to record thermographically; in these regions, the skin temperature is dominated primarily by the skin blood flow. In this respect, a change in skin temperature cannot be directly inferred from the activity of the muscle groups involved. The clearly asymmetrical and significantly different temperature distribution in the abdominal region (m. obliquus) can perhaps be attributed to the fact that the muscles involved in lateral flexion are rather rarely used in everyday life. This may result in a lower training status and consequently in faster fatigue and therefore a higher temperature rise. In the area of the lower back, large muscle strings are active in lateral flexion with the mm. multifidii, which are frequently—and symmetrically—used in everyday life for pelvic stabilization. A resulting better training condition could be a reason for the lack of a clear asymmetric temperature profile in our study. A further possible reason for the findings could also be the way the exercise was performed. Despite control and constant correction during the execution of the treatment, the most common mistake was the rotation of the upper body during lateral trunk extension, which could have caused a shift in fatigue in the direction of the deeper abdominal muscles.

Our study has some limitations. It is known that the body fat distribution influences thermographic reactions [26], which is also indicated by the results of the repeated-measures MANOVA. The sample of subjects contained several subjects with percentual body fat over 25%. Therefore, the effects might have not been visible for the respective subjects due to a thicker layer of subcutaneous adipose tissue, which affects the propagation of heat from the muscle to the surface of the body. The measurement and inclusion of various other possible influencing variables (e.g., body fat distribution, training condition, daily oscillations) in the statistical analysis should be considered for future works. Furthermore, an alternative treatment for introducing asymmetric fatigue could be considered in future works according to the abovementioned limitation. The study was conducted under laboratory environmental conditions; therefore, the results are limited in their application under real-world conditions (e.g., solar radiation leads to changes in skin temperature of individuals undergoing physical work [31]).

## 5. Conclusions

For the abdomen, the clear thermographic differences between the treatment and non-treatment sides suggest the effectiveness of the treatment regarding triggering asymmetrical muscle fatigue.

This may also highlight the potential for the use of thermography data in the context of differential diagnosis for being able to detect muscular imbalances. In the context of asymmetric fatigue, the additional consideration of the spine through analysis of the vertebral orientations (see for example [32]) may add additional insights and should be considered in future works. Furthermore, in order to be able to make a statement about the monitoring ability of the thermography and physical load, it seems interesting to check these results with EMG.

## Figures and Tables

**Table 1:** Anthropometric data of the sample of subjects.

	Min	Max	Mean
**Age (years)**	19	34	22.63 ± 3.91
**Height (cm)**	154.00	189.00	173.36 ± 9.95
**Weight (kg)**	52.60	106.10	71.89 ± 12.97
**Body fat (%)**	6.00	39.40	20.92 ± 9.58
**Muscle mass (kg)**	14.00	52.60	31.48 ± 8.70

**Table 2:** Skin surface temperatures for the measured areas for the treatment and non-treatment side during the three measuring times.

	Pre		Post		Follow-Up	
	M	SD	M	SD	M	SD
Upper treatment side back (Area 1)	31.48	1.06	30.42	1.42	31.44	0.97
Upper non-treatment side back (Area 2)	31.48	1.05	29.88	1.22	31.48	1.04
Lower treatment side back (Area 3)	31.38	1.05	30.04	1.18	31.22	1.06
Lower non-treatment side back (Area 4)	31.43	1.03	29.72	1.13	31.26	1.07
Upper treatment side abdomen (Area 1)	32.01	0.95	30.97	1.23	31.77	1.01
Upper non-treatment side abdomen (Area 2)	31.98	0.92	31.89	1.34	31.72	0.98
Lower treatment side abdomen (Area 3)	31.65	0.89	30.62	1.23	31.36	1.07
Lower non-treatment side abdomen (Area 4)	31.57	0.88	31.57	1.27	31.32	1.04

M: mean; SD: standard deviation.

## Data Availability

The data are not publicly available, as recognition of the subjects based on the thermographic image data cannot be ruled out.
